# Resolvin D1 Alleviates the Lung Ischemia Reperfusion Injury via Complement, Immunoglobulin, TLR4, and Inflammatory Factors in Rats

**DOI:** 10.1007/s10753-016-0364-9

**Published:** 2016-05-04

**Authors:** Qifeng Zhao, Ji Wu, Zhiyong Lin, Qingwang Hua, Weixi Zhang, Leping Ye, Guowei Wu, Jie Du, Jie Xia, Maoping Chu, Xingti Hu

**Affiliations:** The Children’s Department of Cardiovascular and Thoracic Surgery, Children’s Heart Center, The Second Affiliated Hospital and Yuying Children’s Hospital, Institute of Cardiovascular Development and Translational Medicine, Wenzhou Medical University, Wenzhou, 325027 People’s Republic of China; Wuhan Medical & Healthcare Center for Woman and Children, Wuhan, People’s Republic of China; The Children’s Department of Respiration Medicine, The Second Affiliated Hospital and Yuying Children’s Hospital, Wenzhou Medical University, Wenzhou, 325027 People’s Republic of China; The Children’s Department of Cardiovascular Medicine, Children’s Heart Center, the Second Affiliated Hospital and Yuying Children’s Hospital, Institute of Cardiovascular Development and Translational Medicine, Wenzhou Medical University, Wenzhou, 325027 People’s Republic of China

**Keywords:** resolvin, lung ischemia/reperfusion injury, complement, immunoglobulin, inflammation, oxidative stress

## Abstract

Lung ischemia-reperfusion injury (LIRI) is still an unsolved medical issue, which negatively affects the prognosis of many lung diseases. The aim of this study is to determine the effects of RvD1 on LIRI and the potential mechanisms involved. The results revealed that the levels of complement, immunoglobulin, cytokines, sICAM-1, MPO, MDA, CINC-1, MCP-1, ANXA-1, TLR4, NF-κBp65, apoptosis index, and pulmonary permeability index were increased, whereas the levels of SOD, GSH-PX activity, and oxygenation index were decreased in rats with LIRI. Except for ANXA-1, these responses induced by LIRI were significantly inhibited by RvD1 treatment. In addition, LIRI-induced structure damages of lung tissues were also alleviated by RvD1 as shown by H&E staining and transmission electron microscopy. The results suggest that RvD1 may play an important role in protection of LIRI via inhibition of complement, immunoglobulin, and neutrophil activation; down-regulation of TLR4/NF-κB; and the expression of a variety of inflammatory factors.

**Electronic supplementary material:**

The online version of this article (doi:10.1007/s10753-016-0364-9) contains supplementary material, which is available to authorized users.

## Introduction

Lung ischemia–reperfusion injury (LIRI) is still an unsolved medical issue both in research and clinic [1, 2]. LIRI is associated with the lung transplant, extracorporeal circulation, post-enucleation of pulmonary embolism, and pneumonectomy, which could result in pulmonary dysfunction and severe damages [3, 4]. The pathogenesis of LIRI has been studied for many years. In this respect, oxygen radicals, inflammatory mediators, and neutrophils have been identified to play important roles in ischemia–reperfusion injury (IRI) [5]. More recently, LIRI has been considered as a congenital autoimmune reaction [6]. Indeed, ischemia-exposed antigens on the membrane could bind with the toll-like receptor 4 (TLR4) during reperfusion and activate the related immune response [7]. In addition, the ischemic antigen could also bind with the plasma-specific immunoglobulin and form immune complexes to promote the inflammatory reaction and aggravate the lung injury [8].

Identifying effective and safety methods/drugs to reduce the damages of LIRI is still a hot research area. The anti-inflammatory effect of endogenous lipid mediators, such as resolvin (Rv) and lipoxin, has been tested in many studies [9–11]. These specialized pro-resolving mediators have the conserved structures with the synthesized biological functions in host defense, pain, organ protection, and tissue remodeling [12, 13], which could protect several organs such as the brain, lung, kidney, and stomach from IRI [14–18]. However, the effect of Rv on LIRI is still unclear.

In the present study, we aimed to investigate the effect and the related mechanism of resolvin D1 (RvD1) on LIRI in rats. In particular, we studied the effects of RvD1 on the change of complement, immunoglobulin protein, TLR4, nuclear factor kappaB (NF-κB) p65, inflammatory response, oxidative stress, the pathological structure, and the pulmonary function in rats.

## Materials and Methods

### Rat Model of LIRI

All animal protocols were approved by the Institutional Animal Care and Use Committee at Wenzhou Medical University and were consistent with the Guide for the Care and Use of Laboratory Animals (updated (2011) version of the NIH guidelines). Sprague Dawley (SD) male rats (8 weeks old, 200 to 250 g) were fed with a standard diet and maintained in a controlled environment of the animal center at 25 ± 1 °C under a 12-h light–dark cycle.

Rats were anesthetized by an intraperitoneal injection of 10 % chloral hydrate (300 mg/kg^−1^ body weight) and placed in a supine position. The animals were then intubated for artificial ventilation with oxygen using a small animal breathing machine (tidal volume 5 ml, frenquency 70 per min) and electrocardiograph monitor. Thoracotomy was performed at the anterior lateral side of the left fourth intercostal. The muscular layer and pleura were gently dissected to expose the heart and lung. After that, the hilum of left lung was dissociated and the artery clamp was used to pass through the hilum of lung from the upper right to the lower left. The whole clamped left hilum was clearly exposed by slightly stirring up the clamp. Before blocking, heparin was injected intravenously (1 mg/kg body weight). After ischemia for 45 min, the artery clamp was removed (no blocking in sham group) and then reperfusion was started and lasted for 150 min. During the reperfusion time, 0.5 ml normal saline (NS) was injected intravenously every hour to maintain the body fluid. After that, the chest wall was closed, the animal was extubated, and the body temperature was maintained using a warming plate.

### Animal Grouping and Treatments

RvD1 (C_22_H_32_O_5_, 7S,8R, 17S-trihydroxy-4Z, 9E, 11E, 13Z, 15E, 19Z-docosahexaenoicacid; see Supplementary Fig. 1 in the Supplementary Material) was purchased from Cayman Chemical Company, Ann Arbor, USA (cat. number 10012554). Forty-eight SD rats were randomly divided into four groups (12 rats/group) as follows: (1) sham group: no blocking of hilum in left lung; (2) ischemia-reperfusion (IR)-control (C) group: blocking for 45 min and reperfusion for 150 min; (3) IR-NS group: blocking for 45 min, reperfusion for 10 min followed by injection of 2 ml/kg NS by formal vein and continuous reperfusion for 140 min; (4) IR-RV group: blocking for 45 min, reperfusion for 10 min followed by injection of 100 μg/kg RvD1 by formal vein, and continuous reperfusion for 140 min.

### Blood, Bronchoalveolar Lavage Fluid Collection, and Tissue Harvest

Blood samples were collected in each group immediately before thoracotomy (T_1_) or after the experiments (T_2_). In the sham group, T_2_ was obtained after 195 min of the artery clip across the left hilus pulmonis. For all other groups, T_2_ blood samples were obtained after 150 min of reperfusion. Rats were sacrificed after blood collection. The bronchoalveolar lavage fluid (BALF) was then collected by washing the airways of the left lungs three times with a total of 5 ml of phosphate buffer solution through a tracheal cannula (recovery rate >80 %), which was pooled and centrifuged at 3000 rpm/min for 15 min for further use. The left lung tissue of rats was dissected to measure the wet to dry weigh ratio (W/D) value. Other lung tissues were fixed in 4 % paraformaldehyde or frozen in −70 °C refrigerator for further analysis.

### Lung Tissue W/D

About 1 g of lung tissue was measured and named as wet weight. The tissue was then kept in 70 °C electrothermal constant-temperature dry box for 48 h, and the weight of tissue was designed as dry weight. W/D was calculated and analyzed, which can be an indicator of the lung tissue edema.

### BALF Leukocyte Count, BALF Neutrophil Ratio and Pulmonary Permeability Index

Samples of BALF precipitate were analyzed for the number of leukocyte. Through wright staining, the BALF neutrophil ratio was obtained. The supernatant of BALF and blood serum was harvested for total protein analysis using the Bradford method. The ratio of total protein in BALF to the total protein in blood serum was calculated and named as Pulmonary Permeability Index (PPI).

### Oxygenation Index

The arterial blood gas analysis was performed at T_2_, and the ratio of PaO_2_ to FiO_2_ was then obtained as an oxygenation index.

### Lung Tissue Hematoxylin–Eosin Staining

Lung samples obtained at T_2_ were fixed in 4 % paraformaldehyde and subsequently embedded in paraffin. Sections (5 μm thick) were stained with hematoxylin–eosin (HE) using a standard protocol and analyzed by light microscopy.

### Transmission Electron Microscopy

Lung samples were dissected and immediately fixed in 0.1 M phosphate buffer containing 2.5 % glutaraldehyde and 2 % paraformaldehyde for 4 h. The samples were then fixed with 1 % osmium tetroxide for 2 h, dehydrated through a graded ethanol series, and embedded in epoxy resin. Resin-embedded blocks were cut into 60∼80-nm ultrathin sections with an ultramicrotome (PT-XL, RMC, USA). The ultrathin sections were placed on carbon-coated nickel grids and examined with an H-7500 transmission electron microscope (H-7500, Tokyo, Japan).

### Complement, Immune Globulin, Cytokine, and Adhesion Molecule Levels

Blood samples were collected by femoral venipuncture at set time points, before thoracotomy (T_1_) and after reperfusion (T_2_). The serum was then analyzed by an ELISA kit (Boyun Biotech, Shanghai, China) to determine the levels of complements (C1q, C2, C3a, C4, C5a), immune globulin (Ig)M and IgG, cytokines (interleukin (IL)-1β, IL-6, tumor necrosis factor (TNF)-α), and soluble intercellular adhesion molecule (sICAM)-1) in accordance with the manufacturer’s instructions.

### Cytokine-Induced Neutrophil Chemoattractant-1, Monocyte Chemoattractant Protein-1, and Annexin-1 Determination

The lung tissue homogenate was centrifuged, and the supernatant was used to determine the concentration of cytokine-induced neutrophil chemoattractant (CINC)-1, monocyte chemoattractant protein (MCP)-1, and annexin-1 (ANXA-1) by an ELISA kit (Boyun Biotech, Shanghai, China) according to the manufacturer’s instructions.

### Myeloperoxidase, Superoxide Dismutase, Glutathione Peroxidase Activity, and Malondialdehyde Content Determination

The lung tissue myeloperoxidase (MPO) activity was determined on frozen tissue by use of colorimetry assay kits (Jiancheng Bioengineering Institute, Nanjing, China). The lung tissue superoxide dismutase (SOD) and glutathione peroxidase (GSH-PX) activity was determined on frozen tissue using Xanthine Oxidase Assay kits (Jiancheng Bioengineering Institute, Nanjing, China). The malondialdehyde (MDA) content was determined on frozen lung tissue by use of the thiobarbituric acid assay kit (Jiancheng Bioengineering Institute, Nanjing, China).

### Real-Time Quantitative Polymerase Chain Reaction Analysis

Total RNAs of the tissues were extracted using TRIzol Reagent (Invitrogen, USA) according to the manufacturer’s instructions, and the total RNA concentrations were quantified. Subsequently, 500 ng of total RNA was reversed via the complementary DNA (cDNA) synthesis kit (Invitrogen, USA). Real-time quantitative polymerase chain reaction (RT-qPCR) was achieved using the SYBR Green system (Bio-Rad, USA). Amplifications for cDNA samples were carried out using a PCR machine in the following conditions: 95 °C for 90 s, followed by 40 cycles (95 °C for 5 s and 58 °C for 30 s). Primer sequences of TLR4 and NF-κBp65 are as shown on Table 1. The relative quantification of target gene was normalized to GAPDH and calculated using the absolute quantification standard curve method. Melting curve profiles were produced at the end of each PCR so as to confirm the specific transcriptions of amplification. Each sample was analyzed in triplicate.

**Table 1:** Real-Time PCR Primer Sequences

Gene	Forward primer	Reverse primer	Size (bp)
TLR4	5ʹ-TTATCCAGAGCCGTTGGTGT-3ʹ	5ʹ-CCCACTCGAGGTAGGTGTTT-3ʹ	171 bp
NF-κBp65	5ʹ-TTCCTGCTTACGGTGGGATT-3ʹ	5ʹ-CCCCACAT CCTCTTCCTTGT-3ʹ	248 bp
GAPDH	5ʹ-GAGACAGCCGCATCTTCTTG-3ʹ	5ʹ-TGACTGTGCCGTTGAACTTG-3ʹ	224 bp

### Western Blotting Analysis

Lung tissues lysis was obtained using RIPA buffer and then centrifuged to obtain the total protein. Equal amounts of protein (50 μg) were subjected to SDS-PAGE. Gels were transferred to polyvinylidene fluoride (PVDF) membrane. Membrane was blocked with 5 % nonfat dry milk in Tris-buffered saline, 0.1 % Tween 20 (Sigma, USA), and immunoblotting was performed using TLR4 and NF-κBp65 rabbit anti-rat antibody (Cell Signaling Technology, USA) as described by the manufacturer. Anti-β-actin antibody (Santa Cruz Biotechnology, USA) was used as loading control. Blots were then developed by incubation with biotinylated anti-rabbit antibodies (Vector Laboratories, USA) as secondary antibodies, followed by incubation with ABC reagent (GE, USA). Signal was detected using a luminescence kit (GE, USA) and X-ray film.

### TdT-Mediated dUTP Nick End Labeling Assay

Apoptosis was determined by TdT-mediated dUTP nick end labeling (TUNEL) assay with TUNEL test kit (Roche, USA) according to the manufacturer’s instructions. Cells with apoptotic morphologic features as well as with tan or brown nuclei were judged to be apoptotic cells. The five fields of view were automatically selected by the Image-Pro Plus version 5.1 image analysis software. The percentage of apoptosis-positive cells was calculated for each field of view. The mean was calculated to obtain the percentage of apoptotic cells and expressed as apoptotic index (AI). AI (%) = (apoptotic nuclei count / total nucleus count) × 100 %.

### Statistical Analysis

Data are expressed as mean ± standard deviation. Statistical analysis was performed by one-way ANOVA to compare more than two groups or with two-tailed unpaired-sample *t* test to compare two groups. All statistical computations were performed using SPSS version 17.0 (SPSS Inc., Chicago, IL, USA). The significance level was set at *P* < 0.05.

## Results

### The Effects of RvD1 on Lung Tissue W/D, BALF Leukocyte Count, BALF Neutrophil Ratio, PPI, and Oxygenation Index

**Fig. 1:**
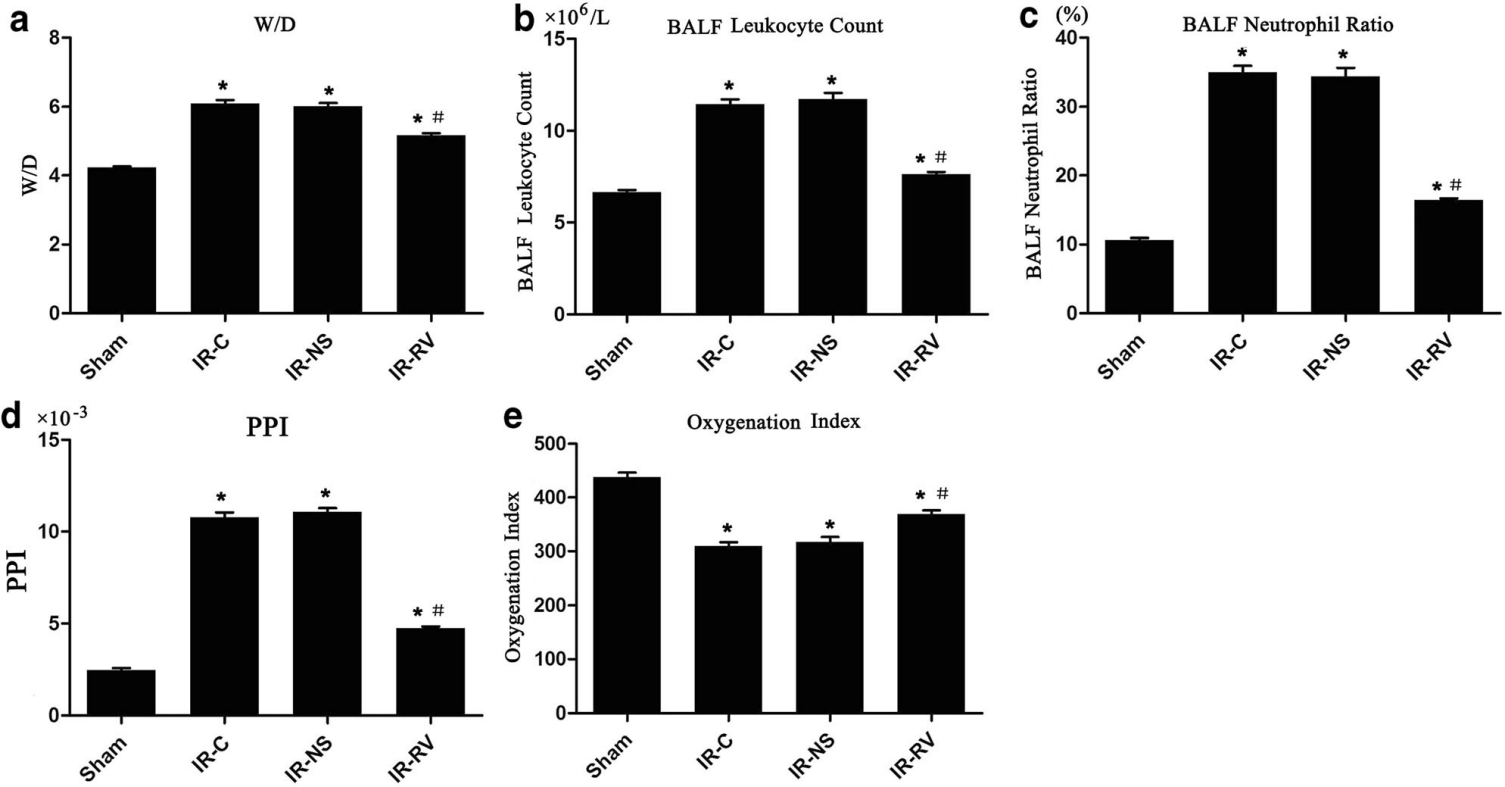
The effects of RvD1 on lung tissue W/D, BALF leukocyte count, BALF neutrophil ratio, PPI, and oxygenation index. Comparison of lung tissue W/D, BALF leukocyte count, BALF neutrophil ratio, PPI, and oxygenation index at T_2_ among all groups. At time point T_2_, lung tissue, BALF, and arterial blood were collected immediately after the IR procedure was completed. W/D, BALF leukocyte count, BALF neutrophil ratio, PPI, and oxygenation index were measured as described in “MATERIALS AND METHODS” section. **a** W/D, **b** BALF leukocyte count, **c** BALF neutrophil ratio, **d** PPI, **e** oxygenation index. Data were expressed as means ± SD and analyzed by ANOVA and unpaired-sample *t* test. *n* = 12 for each group *P < 0.05 for comparisons of IR-C, IR-NS, and IR-RV groups with sham group; #*P* < 0.05 for comparisons of IR-NS and IR-RV groups with IR-C group.

Figure 1 shows the effects of RvD1 on the W/D, BALF leukocyte count, BALF neutrophil ratio, PPI, and oxygenation index in lung tissues. At the time point of T_2_, the values of W/D, BALF leukocyte count, BALF neutrophil ratio, and PPI in IR-C, IR-NS, and IR-RV groups were significantly higher than that in the sham group. Compared to the IR-C group, these values in IR-RV group were significantly reduced (*P* < 0.05), whereas there was no statistic difference between IR-NS group and IR-C group.

In terms of the oxygenation index, the sham group had a higher value than other three groups did. Interestingly, the index in the IR-RV group was also much higher than that in the IR-C group (*P* < 0.05). No difference was found between IR-NS group and IR-C group.

### Pathologic Changes of Lung Tissues

**Fig. 2:**
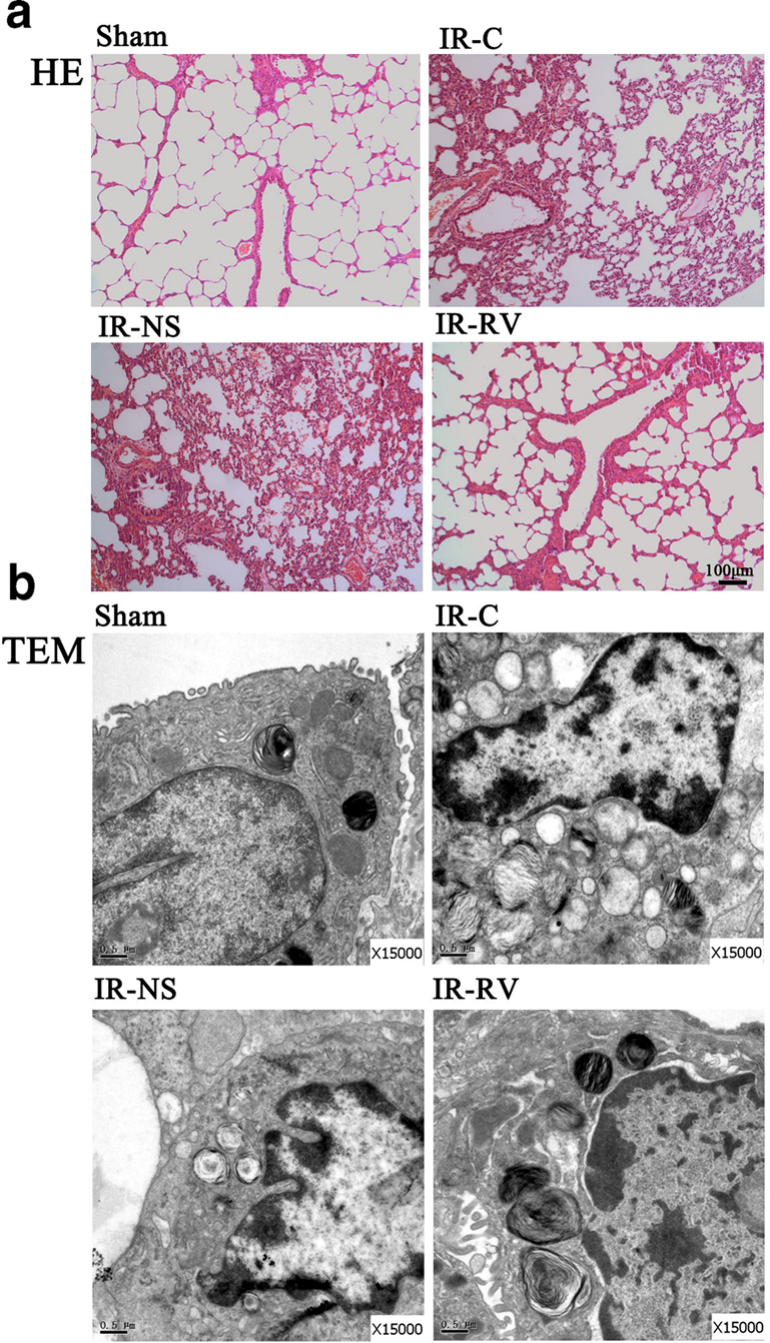
Pathologic changes of lung tissues was examined by H&E staining and TEM. The lung tissue sections were from the following groups of animals: *sham*, aham group; *IR-C*, IR-C group; *IR-NS*, IR-NS group; and *IR-RV*, IR-RV group. **a** H&E staining of lung tissue sections from different groups (*scale bars*: 100 μm, magnification ×100). **b** Transmission electron microscopy (TEM) of lung tissue sections from different groups (magnification ×15000).

To further assess the effect of RvD1 on IRI of the lung, we analyzed pathological changes by H&E staining (Fig. 2a). In the sham group, the pulmonary alveoli and interstitium remain intact with smooth thin alveolar wall and uniform alveolar septal thickness. No apomorphosis, exudation, and neutrophil infiltration were observed. In IR-NS and IR-C groups, damaged alveoli structure and dilated and congestive capillaries could be found. The thickened interstitium was infiltrated with inflammatory cells, while the alveolar lumen was also filled with exudates, red blood cells, and neutrophils. These histological damages in the IR-RV group were inhibited compared with those in IR-NS and IR-C groups. In fact, less neutrophil infiltration and only slight dilatation of the capillaries were observed in the IR-RV group.

The ultrastructure of lung tissue was examined by TEM, as shown in Fig. 2b. In the sham group, the lung tissue ultrastructure was normal with tightly connected pulmonary capillary endothelial cells, intact basement membrane, and integral type I and type II alveolar epithelial cells. The mitochondrial cristae, microvilli, and the lamellar body were clear. However, the ultrastructure was impaired in IR-C and IR-NS groups. The ultrastructure of the lung tissue showed serious abnormalities with swelling pulmonary capillary endothelial cells and mitochondria and shrinking nuclear membrane. Less pinocytosis vesicles can be seen in type I alveolar epithelial cells. Meanwhile, decreased numbers of microvilli and scarce lamellar bodies of the type II alveolar epithelial cells were observed, and a large number of inflammatory cells infiltrated the alveolar septum and capillaries. In the IR-RV group, the decreased injuries were found compared to IR-C and IR-NS groups. The pulmonary capillary endothelial cells and mitochondria exhibited slight swelling. More pinocytosis vesicles were seen in type I alveolar epithelial cells, and the increased number of microvilli and lamellar bodies were seen in type II alveolar epithelial cells. In addition, the alveolar septum had no obvious inflammatory cell infiltration in the IR-RV group.

### The Effects of RvD1 on Serum Levels of C1q, C2, C3a, C4, and C5a

**Fig. 3:**
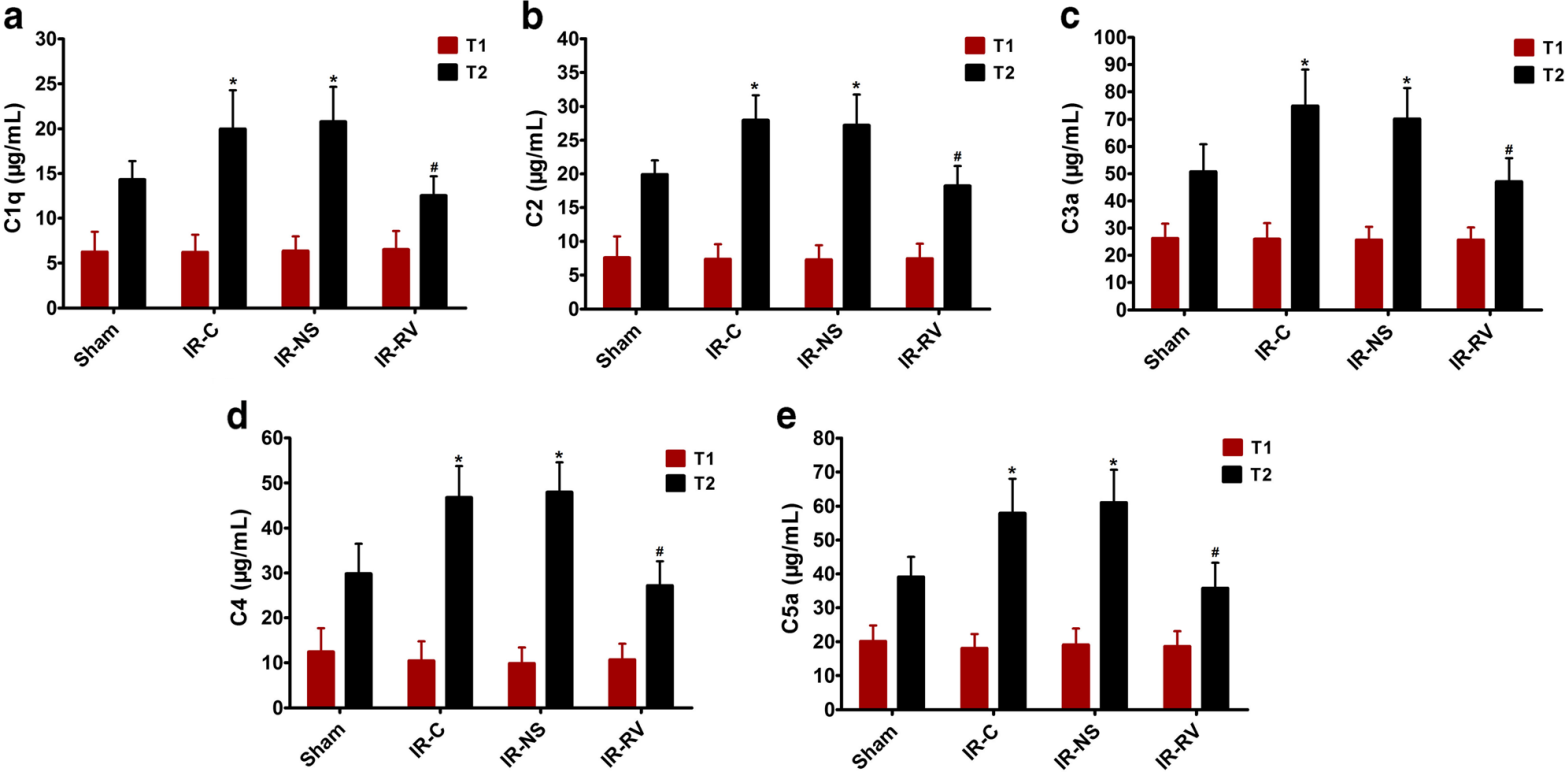
The effects of RvD1 on serum levels of C1q, C2, C3a, C4, and C5a. Comparison of concentrations of serum C1q, C2, C3a, C4, and C5a at T_1_ and T_2_ among all groups. At time point T_1_, blood was collected immediately before thoracotomy. At time point T_2_, blood was collected right after the IR procedure was over. **a** C1q, **b** C2, **c** C3a, **d** C4, **e** C5a. Data were expressed as means ± SD and analyzed by ANOVA and unpaired-sample *t* test. *n* = 12 for each group. *P < 0.05 for comparisons of IR-C, IR-NS, and IR-RV groups with sham group; #*P* < 0.05 for comparisons of IR-NS and IR-RV groups with IR-C group.

The serum levels of C1q, C2, C3a, C4, and C5a were shown in Fig. 3. At T_1_, no difference was found among all the groups. However, at time point of T_2_, the sham group showed obviously lower level of all the complements than the IR-C and IR-NS groups did. Obviously, the IR-RV group exhibited decreased levels of C1q, C2, C3a, C4, and C5a when compared to the IR-C group (*P* < 0.05).

### The Effects of RvD1 on Serum Levels of IgM and IgG

**Fig. 4:**
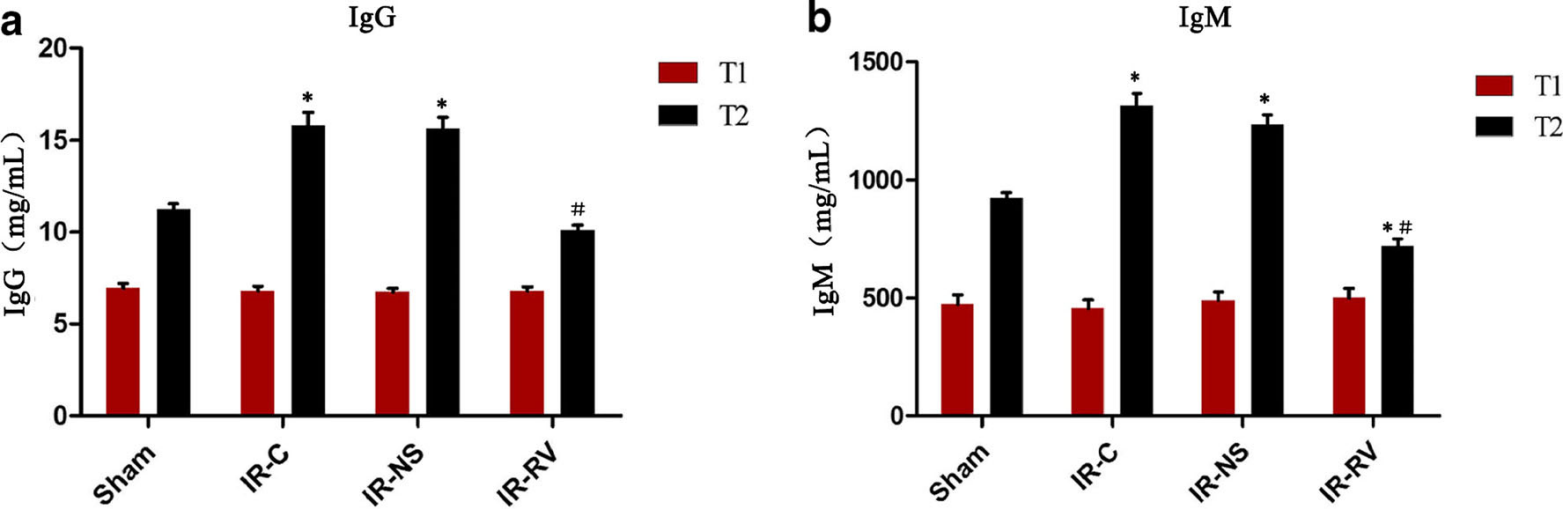
The effects of RvD1 on serum levels of IgG and IgM. Comparison of concentrations of serum IgG and IgM at T_1_ and T_2_ among all groups. At time point T_1_, blood was collected immediately before thoracotomy. At time point T_2_, blood was collected right after the IR procedure was over. **a** IgG, **b** IgM. Data were expressed as means ± SD and analyzed by ANOVA and unpaired-sample *t* test. *n* = 12 for each group. *P < 0.05 for comparisons of IR-C, IR-NS, and IR-RV groups with sham group; #*P* < 0.05 for comparisons of IR-NS and IR-RV groups with IR-C group.

There was no significant difference in IgG and IgM concentrations among all the groups at T_1_ (Fig. 4). At T_2_, the levels of IgG and IgM in IR-C and IR-NS groups were significantly higher than that in the sham group. Compared to the IR-C group, decreased levels of these two immune globulins in the IR-RV group were found (*P* < 0.05).

### The Effects of RvD1 on Serum Levels of IL-1β, IL-6, TNF-α, and sICAM-1

**Fig. 5:**
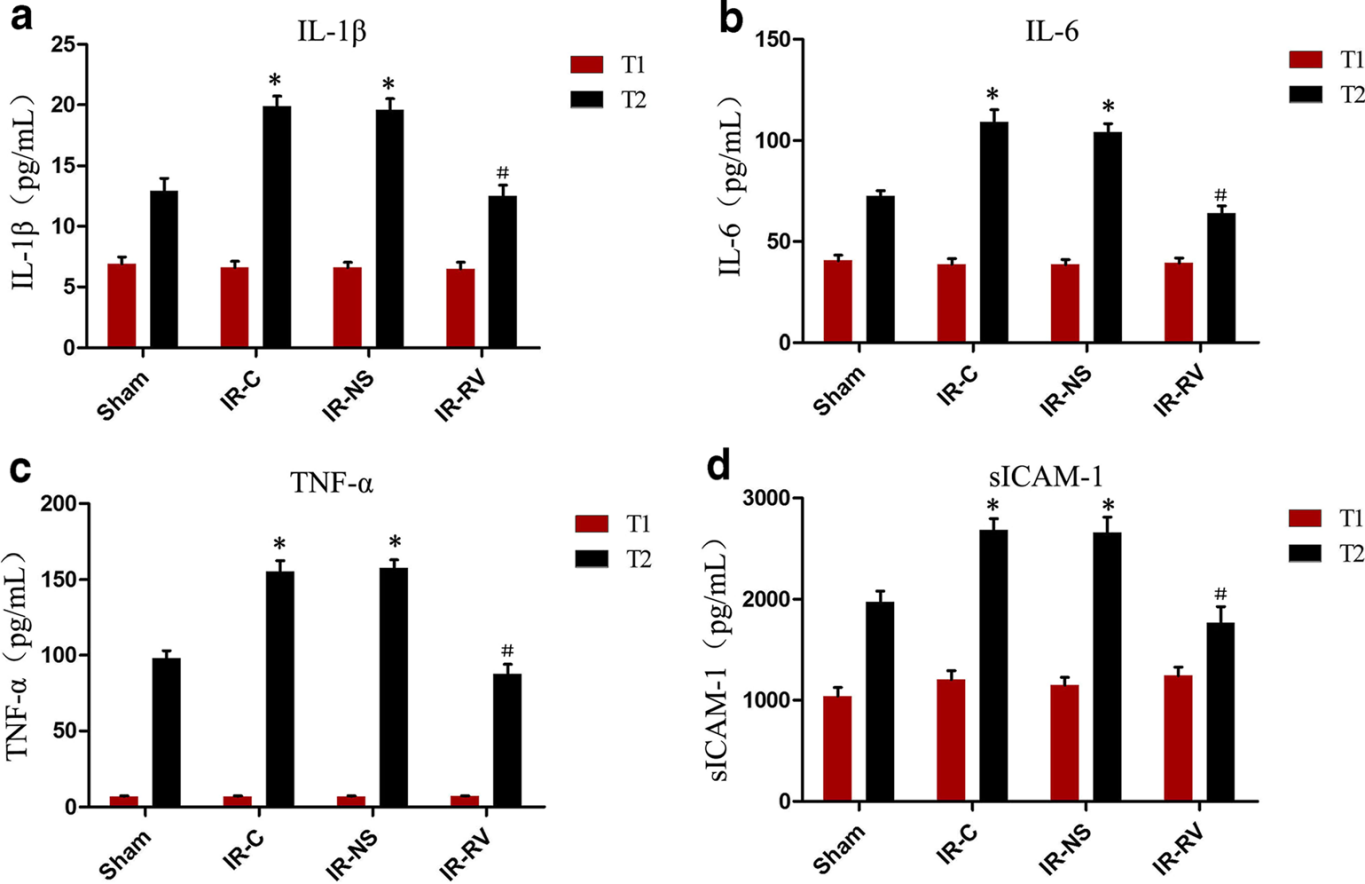
The effects of RvD1 on serum levels of IL-1β, IL-6, TNF-α, and sICAM-1. Comparison of concentrations of serum IL-1β, IL-6, TNF-α, and sICAM-1 at T_1_ and T_2_ among all groups. At time point T_1_, blood was collected immediately before thoracotomy. At time point T_2_, blood was collected right after the IR procedure was over. **a** IL-1β, **b** IL-6, **c** TNF-α, **d** sICAM-1. Data were expressed as means ± SD and analyzed by ANOVA and unpaired-sample *t* test. *n* = 12 for each group. *P < 0.05 for comparisons of IR-C, IR-NS, and IR-RV groups with sham group; #*P* < 0.05 for comparisons of IR-NS and IR-RV groups with IR-C group.

The levels of IL-1β, IL-6, TNF-α, and sICAM-1 showed similar trends to the levels of IgM and IgG. As shown in Fig. 5, we found that no change happened at time T_1_. At T_2_, the IL-1β, IL-6, TNF-α, and sICAM-1 concentrations in IR-C and IR-NS groups were much higher than those in the sham group. A remarkable decrease was found in the IR-RV group when compared to the IR-C group (*P* < 0.05). However, no difference was seen between the IR-C group and the IR-NS group.

### The Effects of RvD1 on CINC-1, MCP-1, and ANXA-1 Content in Lung Tissues

**Fig. 6:**
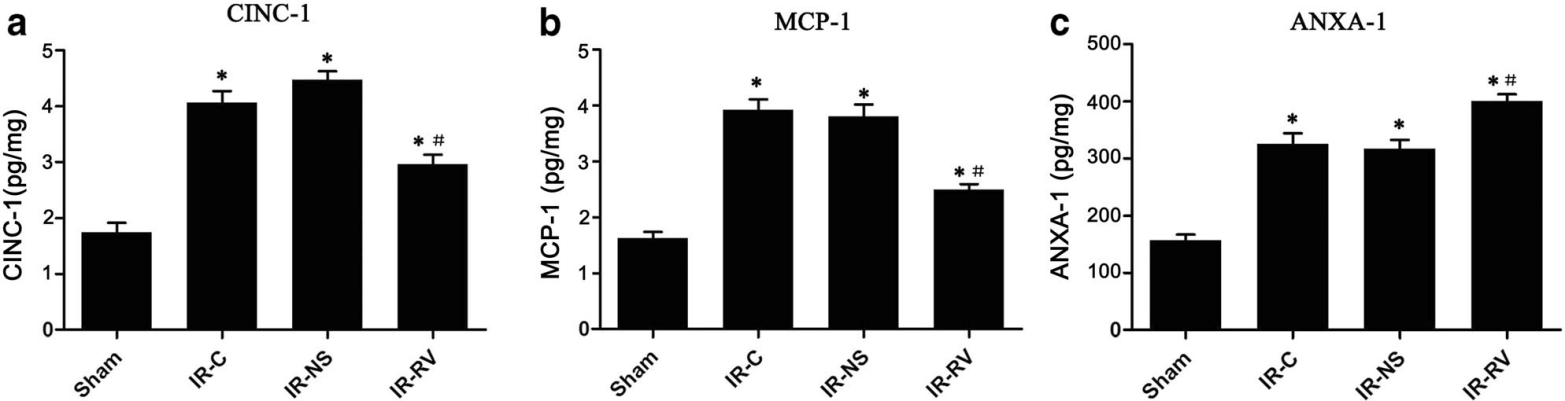
The effects of RvD1 on CINC-1, MCP-1, and ANXA-1 content in lung tissues. Comparison of lung tissue CINC-1, MCP-1, and ANXA-1 content at T_2_ among all groups. At time point T_2_, lung tissue was collected immediately after the IR procedure was completed and kept frozen in liquid nitrogen. CINC-1, MCP-1, and ANXA-1 were measured as described in “MATERIAL AND METHODS” section. **a** CINC-1, **b** MCP-1, **c** ANXA-1. Data were expressed as means ± SD and analyzed by ANOVA and unpaired-sample *t* test. *n* = 12 for each group. *P < 0.05 for comparisons of IR-C, IR-NS, and IR-RV groups with sham group; #*P* < 0.05 for comparisons of IR-NS and IR-RV groups with IR-C group.

All the IR groups showed higher values of CINC-1, MCP-1, and ANXA-1 content than the sham group. As shown in Fig. 6, compared to IR-C, significantly decreased levels of CINC-1 and MCP-1, but elevated level of ANXA-1, in the IR-RV group were observed (*P* < 0.05).

### The Effects of RvD1 on MPO, SOD, and GSH-PX Activity and MDA Production in Lung Tissues

**Fig. 7:**
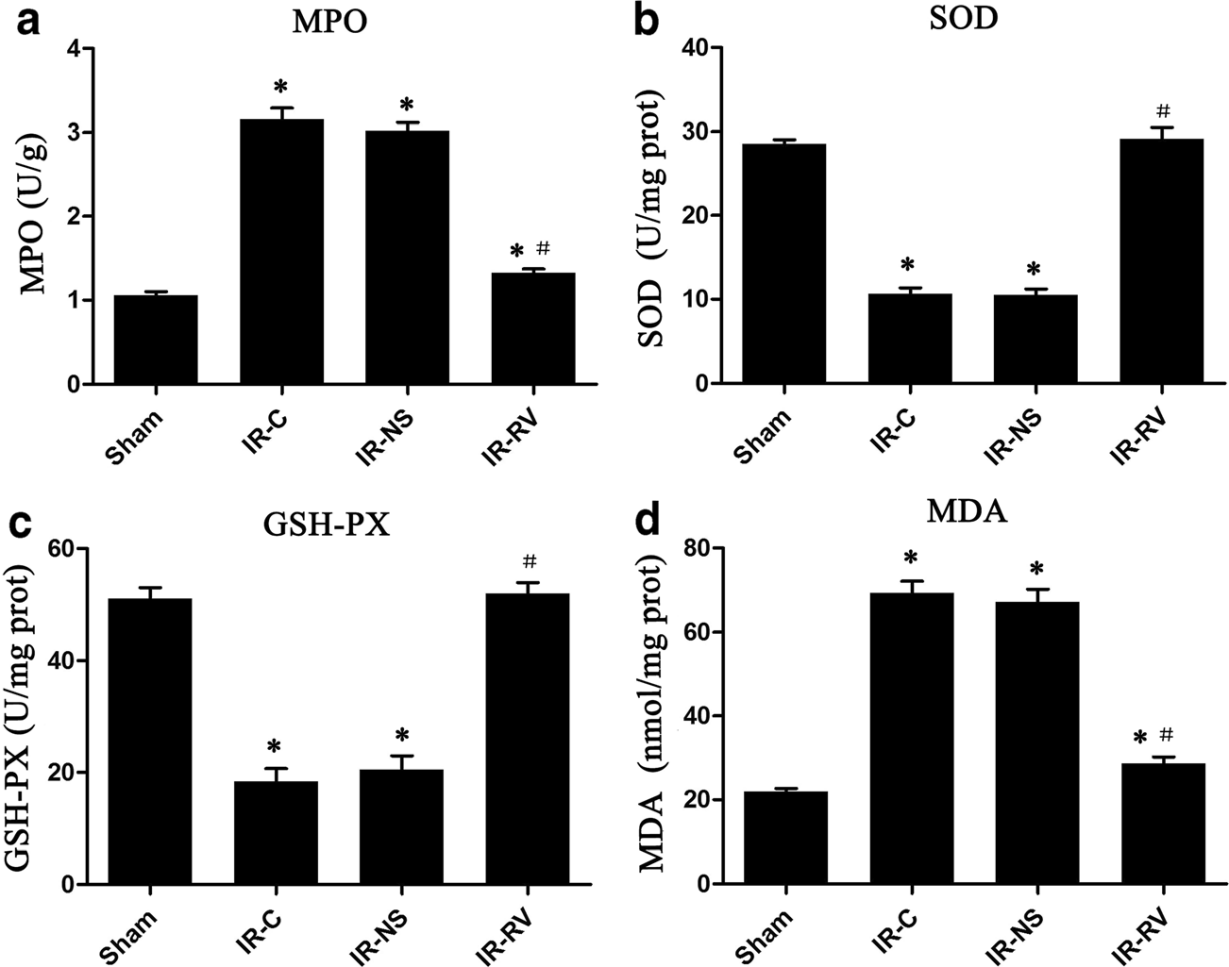
The effects of RvD1 on MPO, SOD, and GSH-PX activity and MDA production in lung tissues. Comparison of lung tissue MPO, SOD, and GSH-PX activity and MDA production at T_2_ among all groups. At time point T_2_, lung tissue was collected immediately after the IR procedure was completed and kept frozen in liquid nitrogen. MPO, SOD, and GSH-PX activity and MDA were measured as described in “MATERIALS AND METHODS” section. **a** MPO, **b** SOD, **c** GSH-PX, **d** MDA. Data were expressed as means ± SD and analyzed by ANOVA and unpaired-sample *t* test. *n* = 12 for each group. *P < 0.05 for comparisons of IR-C, IR-NS, and IR-RV groups with sham group; #*P* < 0.05 for comparisons of IR-NS and IR-RV groups with IR-C group.

The values of MPO, SOD, GSH-PX, and MDA at T_2_ were exhibited in Fig. 7. We found that the values of MPO and MDA in the IR groups were obviously higher than those in the sham group. Among the three IR groups, the IR-RV group showed statistically lower values than did the IR-C group (*P* < 0.05). As for the SOD and GSH-PX levels, the IR-C and IR-NS groups presented decreased status than did the sham group, but no difference was found between the sham group and the IR-RV group. Meanwhile, those two levels were elevated, and significantly higher levels were found in the IR-RV group when compared to the IR-C group (*P* < 0.05).

### The Effects of IR and RvD1 Treatment on TLR4 and NF-κBp65 Gene Expression

**Fig. 8:**
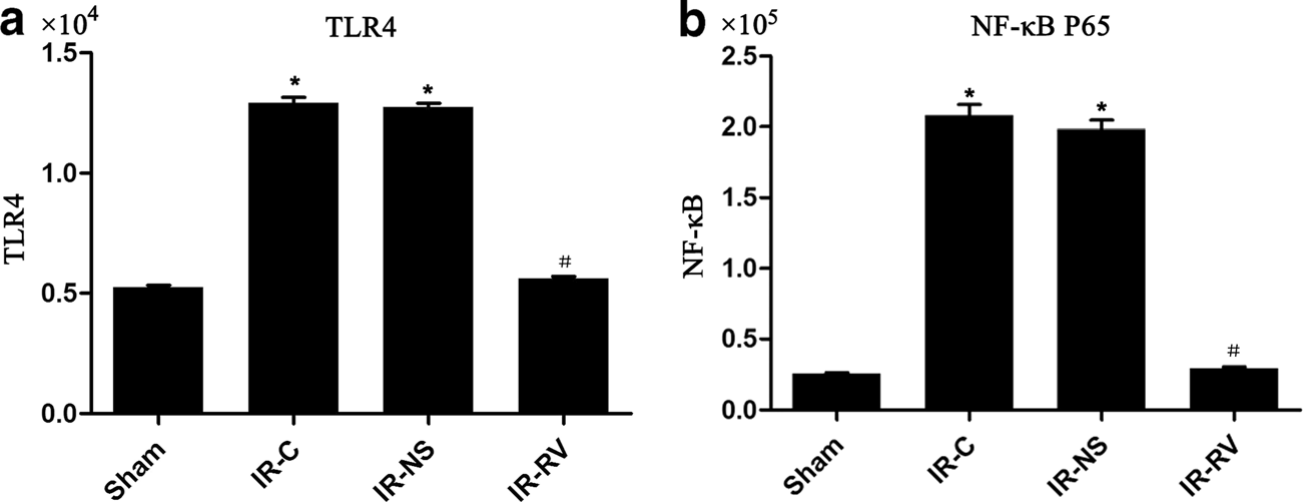
The effects of I/R and RvD1 treatment on TLR4 and NF-κBp65 mRNA expression. Comparison of mRNA levels of TLR4 and NF-κBp65 of lung tissues from rats of different groups at T_2_. Samples were collected right after IR procedure, and the frozen lung tissue measured as described in “MATERIALS AND METHODS” section. **a** TLR4, **b** NF-κBp65. Data were expressed as means ± SD and analyzed by ANOVA and unpaired-sample *t* test. *n* = 12 for each group. *P < 0.05 for comparisons of IR-C, IR-NS, and IR-RV groups with sham group; #*P* < 0.05 for comparisons of IR-NS and IR-RV groups with IR-C group.

The results of the TLR4 and NF-κBp65 expressions at T_2_ in lung tissue were shown in Fig. 8. Compared to the sham group, the messenger RNA (mRNA) levels of TLR4 and NF-κBp65 in IR-C and IR-NS groups were significantly increased (*P* < 0.05). We found that these two genes in the IR-RV group showed a comparable level to the genes in the IR-C group (*P* < 0.05). No change was found between IR-C group and IR-NS group.

### Western Blot Analysis of TLR4 and NF-κBp65 Protein Expression

**Fig. 9:**
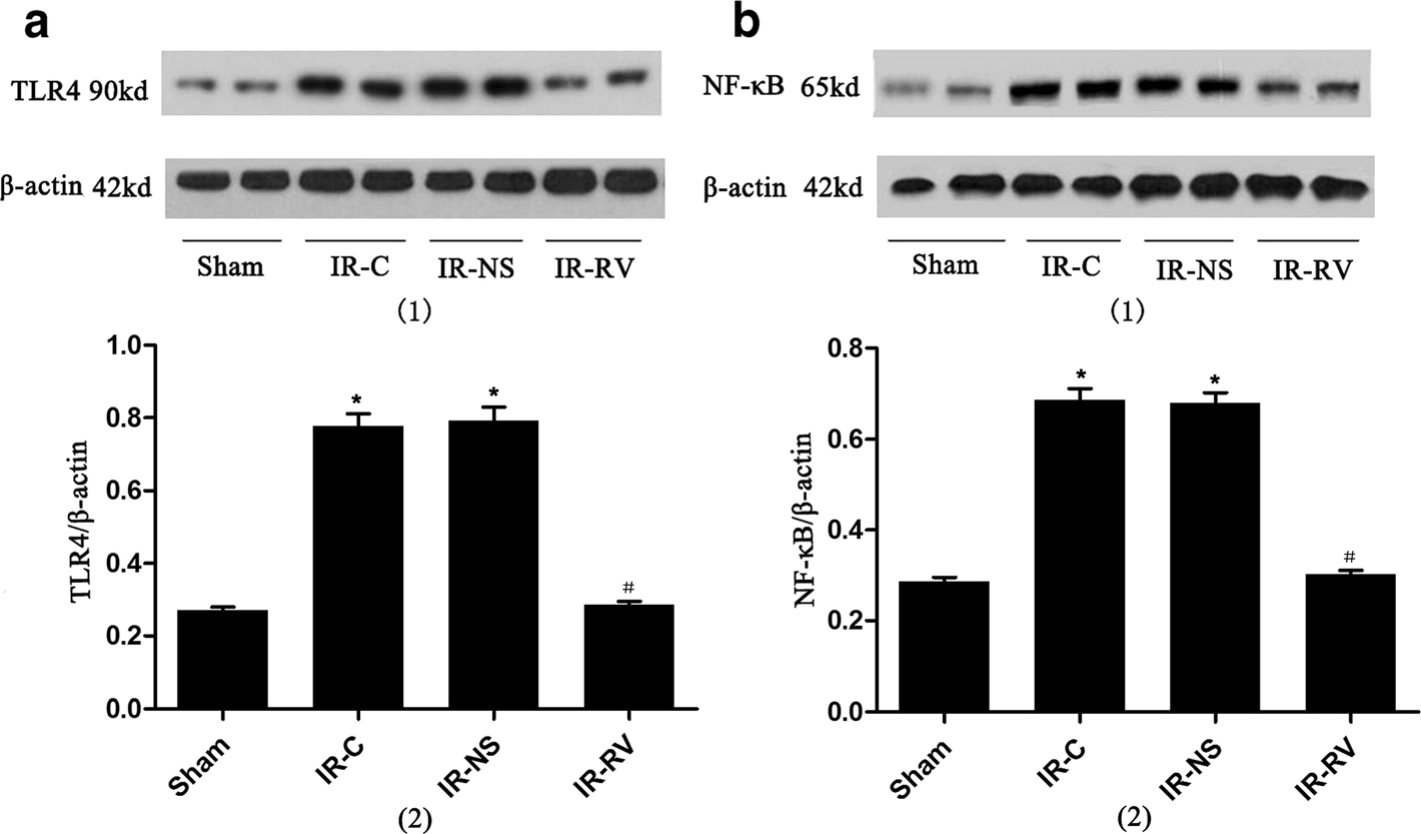
Western blot analysis of TLR4 and NF-κBp65 protein expression. Comparison of protein levels of TLR4 and NF-κBp65 of lung tissues from rats of different groups at T_2_. Samples were collected right after IR procedure was over and western blot was performed as described in “MATERIALS AND METHODS” section. β-Actin was used as a loading control. (*1*) Western blotting: **a** TLR4, **b** NF-κBp65. (*2*) Quantitative data of western blot: **a** TLR4, **b** NF-κBp65. Data were expressed as means ± SD and analyzed by ANOVA and unpaired-sample *t* test. *n* = 12 for each group. *P < 0.05 for comparisons of IR-C, IR-NS, and IR-RV groups with sham group; #*P* < 0.05 for comparisons of IR-NS and IR-RV groups with IR-C group.

The levels of LR4 and NF-κBp65 proteins were remarkably elevated in IR-C and IR-NS groups when compared to the sham group (Fig. 9). Significantly, the decreased levels of TLR4 and NF-κBp65 in the IR-RV group were found compared to the IR-C group (*P* < 0.05).

### Inhibitory Effect of RvD1 on LIRI-Induced Cell Apoptosis

**Fig. 10:**
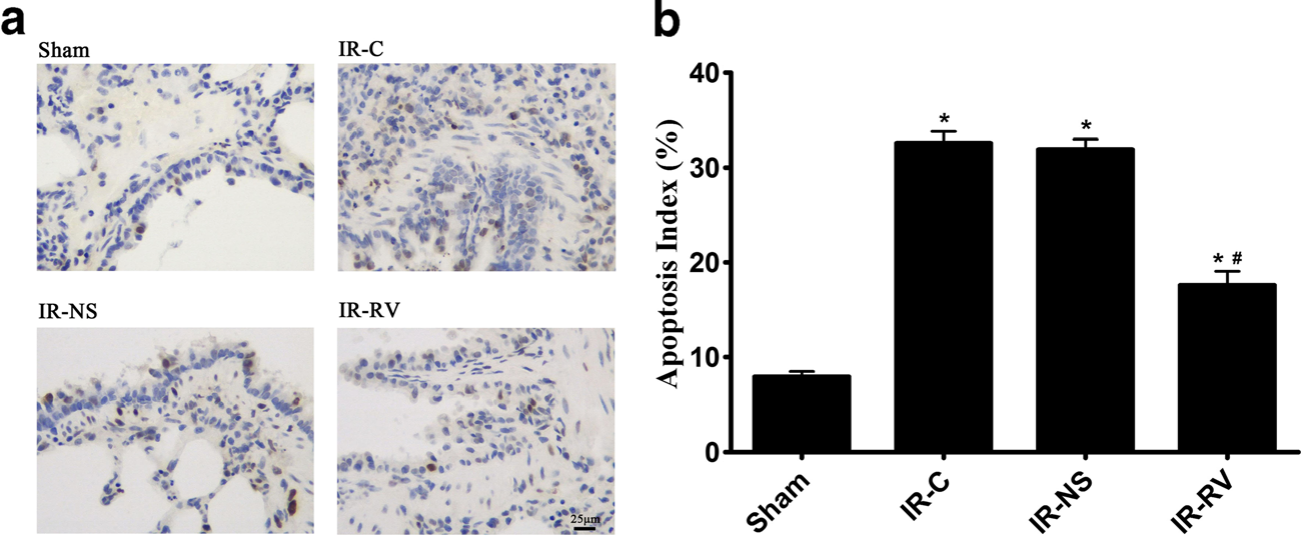
Inhibitory effect of RvD1 on LIRI-induced cell apoptosis. The detection of cell apoptosis in lung tissues from rats of different groups at T_2_. Apoptosis was determined by TUNEL assay according to manufacturer’s instructions as described in “MATERIALS AND METHODS” section. Cells with *apoptotic morphological features* and with *tan or brown nuclei* were judged to be apoptotic cells. **a** TUNEL assay, group sham: a small amount of apoptotic cells in lung tissue; group IR-C and group IR-NS: apoptotic cells in lung tissue increased significantly; group IR-RV: apoptotic cells were between group sham and group IR-C. **b** Apoptosis index (apoptotic nuclei count/total nucleus count) was represented with histogram. Data were expressed as means ± SD and analyzed by ANOVA and unpaired-sample *t* test. *n* = 12 for each group. *P < 0.05 for comparisons of IR-C, IR-NS, and IR-RV groups with sham group; #*P* < 0.05 for comparisons of IR-NS and IR-RV groups with IR-C group.

The effect of RVD1 on lung tissue cell apoptosis at T_2_ was determined and was shown in Fig. 10. The AI of all the three groups were obviously higher than that in the sham group. Among the three IR groups, the index in the IR-RV group was statistically decreased when compared to other two IR groups (*P* < 0.05); however, no difference was found between IR-C and IR-NS group.

## Discussion

Rv is a newly found lipid molecule that can reduce the inflammation and protect the tissue structure by restraining the proliferation of neutrophils, macrophages, and other inflammatory cells [19–21]. In chronic disease such as the inflammatory bowel disease and rheumatoid arthritis, Rv can inhibit humoral immunity and cellular immunity to protect the patients [22, 23]. The animal model of skin infection, gingivitis, peritonitis, and sepsis treated by Rv can relieve the injuries, protect the organs, and increase the survival rates of animals [24]. The research about the protective effect of Rv on the IRI organs, for example, heart and brain, has just been started [15, 25]. However, the effects of RvD1 on LIRI are currently unknown.

The ultrastructure of the lung tissue can finely reflect the pathological changes of the lung injury. In the present study, we observed that the normal lung ultrastructure was damaged by IR, exhibiting swelling pulmonary capillary endothelial cells and mitochondria and shrinking nuclear membrane with a large number of inflammatory cells infiltrating in the alveolar septum and capillaries (Fig. 2b). These abnormalities were also found in the H&E staining sections, which showed the destructive alveoli structure, dilated and congestive capillaries, and inflammatory cells filling the thickened interstitium (Fig. 2a). Moreover, the elevated BALF leukocyte count, BALF neutrophil ratio, W/D, and PPI and the decreased level of oxygenation index were also found in the ischemic reperfusion rats. All these above results are potent evidences of the IRI and pulmonary dysfunctions. Through the treatment of RvD1, less structure changes, neutrophil infiltration, and cell apoptosis; lower PPI; and increased oxygenation index were shown in the IR-RV group, indicating a protective effect of RvD1 on lung tissue.

The occurrence of LIRI has become more frequent in clinical practice during the past decades. In recent years, the research of LIRI has been focused on the following aspects: oxygen radicals and lipid peroxidation [26, 27], excessive inflammatory response caused by neutrophil infiltration [28, 29], body fluid and cellular factors [30], imbalance of intracellular calcium homeostasis [31], and cell apoptosis [32]. Among all the five aspects, the immune reaction might be the most critical factor for the pathogenesis of LIRI. Thrane *et al*. [6] reported that LIRI was a congenital autoimmune reaction. Under the ischemic condition, antigens were exposed and combined with the immune globulin to form the immune complex and activate the complements [8, 33], which could generate a series of bioactive fragments like C3a and C5a and further promote the inflammatory response [34, 35].

LIRI, characterized by nonspecific alveolar damage, hypoxemia, lung edema, and pulmonary hemorrhage, has a direct impact on the prognosis of many related disease, such as the pneumonectomy and the lung transplantation. IR causes lung injury by a variety of reasons. We found that the levels of IgM and IgG and the complements C1q, C2, C3a, C4, and C5a were increased by LIRI. However, after the use of RvD1, they were down-regulated and the damage of lung tissue was alleviated, suggesting that the protective effect of RvD1 was associated with its inhibiting effect on the immune globulin and complements. Tang *et al*. [36] also presented that i.v. administration of either AT-RvD1 or p-RvD1 caused significant decreases in the BALF contents of neutrophils, inflammatory cytokines, chemokines, and complement C5a. These results suggest a new approach to blocking of immune complex-induced inflammation and protecting for lung injury. Other studies also demonstrated that the inhibition of immune globulins and complements can alleviate the reperfusion injury of organs [37–39]. The Cr2−/− [40] and RAG−/− [41] immunodeficient mice showed slight IRI due to their incapability of synthesizing IgM. Besides, the level of IRI was aggravated when the mice were treated with IgM or anti-phospholipid antibodies [42, 43]. In addition, LIRI could activate the complement and produce fragments like C3a and C5a, which are important proinflammatory mediators and chemokines, and could recruit the polymorphonuclear neutrophils (PMNs) to the injury sites. Bless *et al*. [35] reported that the CINC and MIP-2 as well as the complement activation product C5a were required for lung neutrophil recruitment and full induction of lung injury after hindlimb IR in rats. Inhibition of PMN-mediated inflammation can reduce the LIRI [44]. RvD1 is a tissue inflammation-subsided agonist, and its functions are receptor dependent [20, 45]. After LIRI, we applied RvD1 and found less infiltration of PMN in the lung tissue and decreased level of MPO, BALF leukocyte count, and BALF neutrophil ratio, which may be related to the effect of RvD1 on inhibiting complement activation, reducing sICAM-1 and CINC-1 secretion, and promoting ANXA-l expression.

Recent studies have shown that TLR4 plays a key role in the inflammatory cascade of the LIRI [46, 47]. Less damage was found in TLR4-deficient (TLR4 −/−) mice than in wild-type mice (C57BL/6J) in LIRI [48]. NF-κB is a downstream molecule of the TLR4 cascade, which is also important for the autoimmune regulation. The changes of the variety of pro-inflammatory factors, chemokines, adhesion molecules, and enzymes involved in IRI were dependent on the activation of NF-κB [49].

Uncontrolled inflammation usually exists in LIRI. The cellular and molecular changes are controlled by the huge cytokine network. Studies showed that the transcriptional product of NF-κB was the main inflammatory mediator and cytokine in the inflammatory reaction of LIRI, and NF-κB may be the key for the regulation of inflammatory reaction [50]. Thus, NF-κB can affect the LIRI at the transcriptional level by its effects on the inflammatory mediators. Others also showed that LIRI promoted the release of various inflammatory factors, including TNF-α, IL-1, IL-4, IL-6, IL-8, IL-10, and IL-12 [30, 51, 52]. Sharma *et al*. [53] considered that alveolar macrophages produced TNF-α when experiencing LIRI, which further promoted epithelial cells to release the chemokine, such as KC, MCP-1 and MIP-2, RANTES, and IL-6, and aggravated the injury through neutrophils recruiting under the stimulation of chemokine. Ito *et al*. [54] found that LIRI increased the concentrations of TNF-α and CINC-1 of the lung tissue. The CINC-1 of rats, corresponding to the human IL-8 family members in the structure and function, has strong effects on the chemotaxis and activation of the neutrophils [49]. However, ANXA-1 has an opposite effect. *In vitro* and *in vivo* experiments confirmed that through inhibiting the neutrophil adhesion, migration, and the production of proinflammatory mediators and superoxide, endogenous or exogenous ANXA-l can promote the phagocytosis and apoptosis of PMN and further play an important role in anti-inflammation [55, 56].

Our results showed that the cytokines levels of IL-1β, IL-6, TNF-α, and sICAM-1; inflammatory factors levels of CINC-1, MCP-1, and ANXA-1; and the mRNA level and protein expression of TLR4 and NF-κBp65 were up-regulated under LIRI. The results suggest that the TLR4/NF-κB pathway was activated with the increased release of inflammatory factors, adhesion molecules, and chemokine. Moreover, it has been found that Rv participated in the regulation of the NF-κB pathway [57, 58] and NF-κB was the important signaling molecule and intermediate link of Rv biological effect [59]. RvD1 could decrease the NF-κB-phosphorylated p65 nuclear translocation and inhibit expression of the cytokines and chemokines (TNF-α, IL-1β, IL-6, CINC-1, MCP-1, *etc*.) during the inflammatory cascade [60–63]. The inflammatory reaction is one of the vital features of LIRI, and the results of this study were similar with the above previous studies after using RvD1 [60–63]. Specifically, the cytokines and adhesion molecule of IL-1β, IL-6, TNF-α, and sICAM-1 were notably reduced and the inflammatory mediators of CINC-1 and MCP-1 were decreased but with increased levels of ANXA-1, indicating that the inflammatory condition and lung damage were distinctly relieved.

Inflammation is associated with an oxidative stress reaction, which has a positive feedback on inflammation itself [64]. The results of this experiment showed that the level of MDA, representing the degree of lipid peroxidation and the attack of oxygen radicals [65], was significantly increased in LIRI. However, the activity of SOD and GSH-PX, indirectly reflecting the body’s ability to remove the oxygen radicals [66], was significantly reduced. The results suggested that the decreased ability of scavenging free radicals is also related to the lung damage after LIRI. RvD1 can relieve the injury induced by oxidative stress [64] and protect against oxidative stress-initiated inflammation [67]. In our study, RvD1 significantly improved the SOD and GSH-Px activity, indicating that the scavenging ability of the oxygen free radical was enhanced. Meanwhile, the reduced MDA level confirmed that RvD1 can reduce the free radical-induced lung tissue damage and help to restore the body’s oxygen/antioxidant balance. LIRI increased the apoptosis of lung cells [32], and anti-oxidative and anti-inflammatory treatment could reduce the IR-induced lung cell apoptosis [68]. Our results showed that RvD1 can significantly reduce the apoptosis of lung tissue after LIRI, which may be associated with the effect of RvD1 on inhibiting the oxidative stress and inflammation.

RvD1 mainly plays a role in the inflammatory process, but it would not participate in the maintenance of the physiological functions. Therefore, this new type of anti-inflammatory drug may not interfere with the body’s normal physiological activity and cause no obvious adverse reaction. However, the unstable property, short half-life and high price, the unknown dosage and the administrating timing, the frequency, and the delivery ways would limit its application. Thus, the development of the stable analogue of RvD1 may eventually achieve the purpose of clinical use. Further studies are needed to investigate the potential harm of RvD1 to the immune protective function caused by the inhibitory effect on the complement system, especially when suffering from the surgery and other injuries. This experiment did not detect the dynamic changes of the complement system during LIRI, and it is still needed further study to explore the effect of complement after LIRI by using gene-deficient or gene knockout animals. In addition, the lung tissue has a dual blood supply system and can directly obtain oxygen by pulmonary ventilation, which makes LIRI different from other organs’ IRI [69]. The reported models and our animal models of LIRI were achieved by blocking and then loosening the pulmonary hilus, which blocked not only the pulmonary artery but also the bronchus and bronchial artery, leading to the difference from the clinical LIRI. Much effort is still needed to improve the animal model of LIRI and make it closer to clinical practice.

## Conclusions

In conclusion, RvD1 is able to restrain the serum levels of complements and immunoglobulin; inhibit the neutrophil activation; down-regulate the mRNA and protein expression of TLR4 and NF-κB P65; and inhibit the expression of a variety of inflammatory cytokines, chemotactic factors, and adhesion molecules to restore the oxidation/antioxidation balance when suffering from LIRI. Through those effects, the apoptosis and ultrastructure of lung tissue can be protected, resulting in reduced lung injury and improved lung function.

## ELECTRONIC SUPPLEMENTARY MATERIAL

Below is the link to the electronic supplementary material.

Supplementary Fig. 1 Chemical Structure of RvD1 (PDF 107 kb)

## Abbreviations

LIRI: Lung ischemia-reperfusion injury
IRI: Ischemia–reperfusion injury
IR: Ischemia–reperfusion
Rv: Resolvin
SD: Sprague Dawley
BALF: Bronchoalveolar lavage fluid
HE: Hematoxylin–eosin
TEM: Transmission electron microscopy
W: Wet weight
D: Dry weight
PPI: Pulmonary permeability index
Ig: Immune globulin
IL: Interleukin
TNF: Tumor necrosis factor
sICAM: Soluble intercellular adhesion molecule
CINC: Cytokine-induced neutrophil chemoattractant
MCP: Monocyte chemoattractant protein
ANXA-1: Annexin-1
MPO: Myeloperoxidase
GSH-PX: Glutathione peroxidase
SOD: Superoxide dismutase
MDA: Malondialdehyde
RT-qPCR: Real-time quantitative polymerase chain reaction
WB: Western blotting
TLR4: Toll-like receptor 4
NF-κB: Nuclear factor kappaB
TUNEL: TdT-mediated dUTP nick end labeling
AI: Apoptosis index
PMN: Polymorphonuclear neutrophils
PVDF: Polyvinylidene fluoride
NS: Normal saline

## References

[1] Langer F, Schramm R, Bauer M, Tscholl D, Kunihara T, Schafers HJ (2004). Cytokine response to pulmonary thromboendarterectomy. Chest.

[2] Grichnik KP, D’Amico TA (2004). Acute lung injury and acute respiratory distress syndrome after pulmonary resection. Seminars in Cardiothoracic and Vascular Anesthesia.

[3] Ng CS, Wan S, Yim AP, Arifi AA (2002). Pulmonary dysfunction after cardiac surgery. Chest.

[4] van der Kaaij NP, Kluin J, Haitsma JJ, den Bakker MA, Lambrecht BN, Lachmann B, de Bruin RW, Bogers AJ (2008). Ischemia of the lung causes extensive long-term pulmonary injury: an experimental study. Respiratory Research.

[5] de Perrot M, Liu M, Waddell TK, Keshavjee S (2003). Ischemia-reperfusion-induced lung injury. American Journal of Respiratory and Critical Care Medicine.

[6] Thrane AS, Skehan JD, Thrane PS (2007). A novel interpretation of immune redundancy and duality in reperfusion injury with important implications for intervention in ischaemic disease. Medical Hypotheses.

[7] Beutler B (2004). Inferences, questions and possibilities in Toll-like receptor signalling. Nature.

[8] Zhang M, Austen WG, Chiu I, Alicot EM, Hung R, Ma M, Verna N, Xu M, Hechtman HB, Moore FD (2004). Identification of a specific self-reactive IgM antibody that initiates intestinal ischemia/reperfusion injury. Proceedings of the National Academy of Sciences of the United States of America.

[9] Zhang MJ, Spite M (2012). Resolvins: anti-inflammatory and proresolving mediators derived from omega-3 polyunsaturated fatty acids. Annual Review of Nutrition.

[10] Qu Q, Xuan W, Fan GH (2015). Roles of resolvins in the resolution of acute inflammation. Cell Biology International.

[11] Serhan CN, Chiang N, Van Dyke TE (2008). Resolving inflammation: dual anti-inflammatory and pro-resolution lipid mediators. Nature Reviews Immunology.

[12] Fierro IM, Serhan CN (2001). Mechanisms in anti-inflammation and resolution: the role of lipoxins and aspirin-triggered lipoxins. Brazilian Journal of Medical and Biological Research.

[13] Serhan CN (2014). Pro-resolving lipid mediators are leads for resolution physiology. Nature.

[14] Keyes KT, Ye Y, Lin Y, Zhang C, Perez-Polo JR, Gjorstrup P, Birnbaum Y (2010). Resolvin E1 protects the rat heart against reperfusion injury. American Journal of Physiology. Heart and Circulatory Physiology.

[15] Gilbert K, Bernier J, Godbout R, Rousseau G (2014). Resolvin D1, a metabolite of omega-3 polyunsaturated fatty acid, decreases post-myocardial infarct depression. Marine Drugs.

[16] Zhao Q, Shao L, Hu X, Wu G, Du J, Xia J, Qiu H (2013). Lipoxin a4 preconditioning and postconditioning protect myocardial ischemia/reperfusion injury in rats. Mediators of Inflammation.

[17] Zhao Q, Hu X, Shao L, Wu G, Du J, Xia J (2014). LipoxinA4 attenuates myocardial ischemia reperfusion injury via a mechanism related to downregulation of GRP-78 and caspase-12 in rats. Heart and Vessels.

[18] Wu L, Miao S, Zou LB, Wu P, Hao H, Tang K, Zeng P, Xiong J, Li HH, Wu Q (2012). Lipoxin A4 inhibits 5-lipoxygenase translocation and leukotrienes biosynthesis to exert a neuroprotective effect in cerebral ischemia/reperfusion injury. Journal of Molecular Neuroscience.

[19] Navarro-Xavier RA, Newson J, Silveira VL, Farrow SN, Gilroy DW, Bystrom J (2010). A new strategy for the identification of novel molecules with targeted proresolution of inflammation properties. Journal of Immunology.

[20] Schwab JM, Chiang N, Arita M, Serhan CN (2007). Resolvin E1 and protectin D1 activate inflammation-resolution programmes. Nature.

[21] Jin Y, Arita M, Zhang Q, Saban DR, Chauhan SK, Chiang N, Serhan CN, Dana R (2009). Anti-angiogenesis effect of the novel anti-inflammatory and pro-resolving lipid mediators. Investigative Ophthalmology & Visual Science.

[22] Bento AF, Claudino RF, Dutra RC, Marcon R, Calixto JB (2011). Omega-3 fatty acid-derived mediators 17(R)-hydroxy docosahexaenoic acid, aspirin-triggered resolvin D1 and resolvin D2 prevent experimental colitis in mice. Journal of Immunology.

[23] Giera M, Ioan-Facsinay A, Toes R, Gao F, Dalli J, Deelder AM, Serhan CN, Mayboroda OA (2012). Lipid and lipid mediator profiling of human synovial fluid in rheumatoid arthritis patients by means of LC-MS/MS. Biochimica et Biophysica Acta.

[24] Weylandt KH, Chiu CY, Gomolka B, Waechter SF, Wiedenmann B (2012). Omega-3 fatty acids and their lipid mediators: towards an understanding of resolvin and protectin formation. Prostaglandins & Other Lipid Mediators.

[25] Marcheselli VL, Hong S, Lukiw WJ, Tian XH, Gronert K, Musto A, Hardy M, Gimenez JM, Chiang N, Serhan CN (2003). Novel docosanoids inhibit brain ischemia-reperfusion-mediated leukocyte infiltration and pro-inflammatory gene expression. Journal of Biological Chemistry.

[26] Wu SY, Tang SE, Ko FC, Wu GC, Huang KL, Chu SJ (2015). Valproic acid attenuates acute lung injury induced by ischemia-reperfusion in rats. Anesthesiology.

[27] Chen W, Zheng G, Yang S, Ping W, Fu X, Zhang N, Wang DW, Wang J (2014). CYP2J2 and EETs protect against oxidative stress and apoptosis *in vivo* and *in vitro* following lung ischemia/reperfusion. Cellular Physiology and Biochemistry.

[28] Deng C, Zhai Z, Wu D, Lin Q, Yang Y, Yang M, Ding H, Cao X, Zhang Q, Wang C (2015). Inflammatory response and pneumocyte apoptosis during lung ischemia-reperfusion injury in an experimental pulmonary thromboembolism model. Journal of Thrombosis and Thrombolysis.

[29] Jiang L, Li L, Shen J, Qi Z, Guo L (2014). Effect of dexmedetomidine on lung ischemiareperfusion injury. Molecular Medicine Reports.

[30] Zhu B, Yang JR, Chen SF, Jiang YQ (2014). The attenuation of lung ischemia reperfusion injury by oxymatrine. Cell Biochemistry and Biophysics.

[31] Gennai S, Pison C, Briot R (2014). Ischemia-reperfusion injury after lung transplantation. Presse Medicale.

[32] Zhang C, Guo Z, Liu H, Shi Y, Ge S (2015). Influence of levosimendan postconditioning on apoptosis of rat lung cells in a model of ischemia- reperfusion injury. PLoS ONE.

[33] Weiser MR, Williams JP, Moore FD, Kobzik L, Ma M, Hechtman HB, Carroll MC (1996). Reperfusion injury of ischemic skeletal muscle is mediated by natural antibody and complement. Journal of Experimental Medicine.

[34] Guo RF, Ward PA (2005). Role of C5a in inflammatory responses. Annual Review of Immunology.

[35] Bless NM, Warner RL, Padgaonkar VA, Lentsch AB, Czermak BJ, Schmal H, Friedl HP, Ward PA (1999). Roles for C-X-C chemokines and C5a in lung injury after hindlimb ischemia-reperfusion. American Journal of Physiology.

[36] Tang H, Liu Y, Yan C, Petasis NA, Serhan CN, Gao H (2014). Protective actions of aspirin-triggered (17R) resolvin D1 and its analogue, 17R-hydroxy-19-para-fluorophenoxy-resolvin D1 methyl ester, in C5a- dependent IgG immune complex-induced inflammation and lung injury. Journal of Immunology.

[37] Zhang M, Alicot EM, Chiu I, Li J, Verna N, Vorup-Jensen T, Kessler B, Shimaoka M, Chan R, Friend D (2006). Identification of the target self-antigens in reperfusion injury. Journal of Experimental Medicine.

[38] Anderson J, Fleming SD, Rehrig S, Tsokos GC, Basta M, Shea-Donohue T (2005). Intravenous immunoglobulin attenuates mesenteric ischemia-reperfusion injury. Clinical Immunology.

[39] Wada K, Montalto MC, Stahl GL (2001). Inhibition of complement C5 reduces local and remote organ injury after intestinal ischemia/reperfusion in the rat. Gastroenterology.

[40] Reid RR, Woodcock S, Shimabukuro-Vornhagen A, Austen WG, Kobzik L, Zhang M, Hechtman HB, Moore FD, Carroll MC (2002). Functional activity of natural antibody is altered in Cr2-deficient mice. Journal of Immunology.

[41] Austen WG, Kobzik L, Carroll MC, Hechtman HB, Moore FD (2003). The role of complement and natural antibody in intestinal ischemia-reperfusion injury. International Journal of Immunopathology and Pharmacology.

[42] Austen WG, Zhang M, Chan R, Friend D, Hechtman HB, Carroll MC, Moore FD (2004). Murine hindlimb reperfusion injury can be initiated by a self-reactive monoclonal IgM. Surgery.

[43] Fleming SD, Egan RP, Chai C, Girardi G, Holers VM, Salmon J, Monestier M, Tsokos GC (2004). Anti-phospholipid antibodies restore mesenteric ischemia/reperfusion-induced injury in complement receptor 2/ complement receptor 1-deficient mice. Journal of Immunology.

[44] Suzuki S, Sugawara T, Tabata T, Oishi H, Niikawa H, Kondo T (2007). Sivelestat reduces reperfusion injury of lungs harvested from endotoxin-primed rats by inhibition of neutrophil-mediated inflammation. Journal of Heart and Lung Transplantation.

[45] Norling LV, Dalli J, Flower RJ, Serhan CN, Perretti M (2012). Resolvin D1 limits polymorphonuclear leukocyte recruitment to inflammatory loci: receptor-dependent actions. Arteriosclerosis, Thrombosis, and Vascular Biology.

[46] Merry HE, Phelan P, Doak MR, Zhao M, Hwang B, Mulligan MS (2015). Role of toll-like receptor-4 in lung ischemia-reperfusion injury. Annals of Thoracic Surgery.

[47] Zhou Z, Zhu X, Chen J, Yang S, Sun R, Yang G (2014). The interaction between Toll-like receptor 4 signaling pathway and hypoxia-inducible factor 1alpha in lung ischemia-reperfusion injury. Journal of Surgical Research.

[48] Shimamoto A, Pohlman TH, Shomura S, Tarukawa T, Takao M, Shimpo H (2006). Toll-like receptor 4 mediates lung ischemia-reperfusion injury. Annals of Thoracic Surgery.

[49] Ishii H, Ishibashi M, Takayama M, Nishida T, Yoshida M (2000). The role of cytokine-induced neutrophil chemoattractant-1 in neutrophil-mediated remote lung injury after intestinal ischaemia/reperfusion in rats. Respirology.

[50] Linfert D, Chowdhry T, Rabb H (2009). Lymphocytes and ischemia- reperfusion injury. Transplantation Reviews (Orlando, Fla.).

[51] Gao W, Zhao J, Kim H, Xu S, Chen M, Bai X, Toba H, Cho HR, Zhang H, Keshavjeel S (2014). alpha1-Antitrypsin inhibits ischemia reperfusion-induced lung injury by reducing inflammatory response and cell death. Journal of Heart and Lung Transplantation.

[52] Tomasdottir H, Hjartarson H, Ricksten A, Wasslavik C, Bengtsson A, Ricksten SE (2003). Tumor necrosis factor gene polymorphism is associated with enhanced systemic inflammatory response and increased cardiopulmonary morbidity after cardiac surgery. Anesthesia and Analgesia.

[53] Sharma AK, Fernandez LG, Awad AS, Kron IL, Laubach VE (2007). Proinflammatory response of alveolar epithelial cells is enhanced by alveolar macrophage-produced TNF-alpha during pulmonary ischemia-reperfusion injury. American Journal of Physiology. Lung Cellular and Molecular Physiology.

[54] Ito K, Shimada J, Kato D, Toda S, Takagi T, Naito Y, Yoshikawa T, Kitamura N (2004). Protective effects of preischemic treatment with pioglitazone, a peroxisome proliferator-activated receptor-gamma ligand, on lung ischemia-reperfusion injury in rats. European Journal of Cardio-Thoracic Surgery.

[55] Perretti M, D’Acquisto F (2009). Annexin A1 and glucocorticoids as effectors of the resolution of inflammation. Nature Reviews Immunology.

[56] Guido BC, Zanatelli M, Tavares-de-Lima W, Oliani SM, Damazo AS (2013). Annexin-A1 peptide down-regulates the leukocyte recruitment and up-regulates interleukin-10 release into lung after intestinal ischemia- reperfusion in mice. Journal of Inflammation (London).

[57] Herrera BS, Ohira T, Gao L, Omori K, Yang R, Zhu M, Muscara MN, Serhan CN, Van Dyke TE, Gyurko R (2008). An endogenous regulator of inflammation, resolvin E1, modulates osteoclast differentiation and bone resorption. British Journal of Pharmacology.

[58] Ishida T, Yoshida M, Arita M, Nishitani Y, Nishiumi S, Masuda A, Mizuno S, Takagawa T, Morita Y, Kutsumi H (2010). Resolvin E1, an endogenous lipid mediator derived from eicosapentaenoic acid, prevents dextran sulfate sodium-induced colitis. Inflammatory Bowel Diseases.

[59] Wang B, Gong X, Wan JY, Zhang L, Zhang Z, Li HZ, Min S (2011). Resolvin D1 protects mice from LPS-induced acute lung injury. Pulmonary Pharmacology & Therapeutics.

[60] Eickmeier O, Seki H, Haworth O, Hilberath JN, Gao F, Uddin M, Croze RH, Carlo T, Pfeffer MA, Levy BD (2013). Aspirin-triggered resolvin D1 reduces mucosal inflammation and promotes resolution in a murine model of acute lung injury. Mucosal Immunology.

[61] Hsiao HM, Thatcher TH, Levy EP, Fulton RA, Owens KM, Phipps RP, Sime PJ (2014). Resolvin D1 attenuates polyinosinic-polycytidylic acid-induced inflammatory signaling in human airway epithelial cells via TAK1. Journal of Immunology.

[62] Weylandt KH, Krause LF, Gomolka B, Chiu CY, Bilal S, Nadolny A, Waechter SF, Fischer A, Rothe M, Kang JX (2011). Suppressed liver tumorigenesis in fat-1 mice with elevated omega-3 fatty acids is associated with increased omega-3 derived lipid mediators and reduced TNF-alpha. Carcinogenesis.

[63] Naidu BV, Farivar AS, Woolley SM, Grainger D, Verrier ED, Mulligan MS (2004). Novel broad-spectrum chemokine inhibitor protects against lung ischemia-reperfusion injury. Journal of Heart and Lung Transplantation.

[64] Wang L, Yuan R, Yao C, Wu Q, Christelle M, Xie W, Zhang X, Sun W, Wang H, Yao S (2014). Effects of resolvin D1 on inflammatory responses and oxidative stress of lipopolysaccharide-induced acute lung injury in mice. Chinese Medical Journal.

[65] Urso ML, Clarkson PM (2003). Oxidative stress, exercise, and antioxidant supplementation. Toxicology.

[66] Li YW, Zhang Y, Zhang L, Li X, Yu JB, Zhang HT, Tan BB, Jiang LH, Wang YX, Liang Y (2014). Protective effect of tea polyphenols on renal ischemia/reperfusion injury via suppressing the activation of TLR4/NF-kappaB p65 signal pathway. Gene.

[67] Spite M, Summers L, Porter TF, Srivastava S, Bhatnagar A, Serhan CN (2009). Resolvin D1 controls inflammation initiated by glutathione-lipid conjugates formed during oxidative stress. British Journal of Pharmacology.

[68] Cao QF, Qu MJ, Yang WQ, Wang DP, Zhang MH, Di SB (2015). Ischemia postconditioning preventing lung ischemia-reperfusion injury. Gene.

[69] Adams JM (2003). Ways of dying: multiple pathways to apoptosis. Genes & Development.

